# Profile Evaluation of Patients with Cleft Lip and Palate Undergoing Surgery at a Reference Center in Rio de Janeiro, Brazil

**DOI:** 10.1155/2012/620302

**Published:** 2012-12-04

**Authors:** Diogo Franco, Marcella Iani, Ronaldo Passalini, Ivan Demolinari, Marcio Arnaut, Talita Franco

**Affiliations:** ^1^Plastic Surgery Division, HUCFF-UFRJ, Rua Ramon Franco, 98 Urca, 22290-290 Rio de Janeiro, RJ, Brazil; ^2^Medical School, HUCFF-UFRJ, 22290-290 Rio de Janeiro, RJ, Brazil

## Abstract

In Brazil, the classic timeline for operating on cleft lip and palate is three months old for cheiloplasty and is 12 to 18 months old for palatoplasty. As from Brazilian treatment centers are usually located in major cities, patients living in more remote areas are often unable to receive treatment at the ideal ages. Data were analyzed retrospectively on 45 patients with cleft lip and/or palate, consecutively operated at the Reference Center, Rio de Janeiro Federal University, Brazil. Particularly noteworthy among these data are gender, clinical presentation, operations performed, age of surgery, and the distance between their homes and the hospital. The average age of patients undergoing primary cheiloplasty was 9.4 months, with primary palatoplasties performed at an average age of 7.2 years. As 67% of these patients lived in other towns, they encountered difficulties in seeking and continuing specialized care. Despite attempts to decentralize cleft palate care in Brazil, suitable conditions are not yet noted for following the treatment protocols in a full and adequate manner.

## 1. Introduction

Cleft lip and palate are among the most common facial congenital anomalies [1]. In addition to adverse effects on appearance, they also cause problems with speech, occlusion, facial growth, and otological disorders.

The treatment for this pathology is complex, extending from birth through to the end of puberty and involving practitioners in a broad range of specialties, including physicians, dentists, and speech therapists. Treatment is intended mainly to maintain nasal respiration and foster adequate facial growth, closing the cleft lip and providing tongue-palate coaptation and orofacial tonus. Patients are generally treated in specialized centers with adequate experience.

In Brazil, the classic timeline for operating on cleft lips and palates consists of cheiloplasty at around three to four months old, with palatoplasty at 12 to 18 months old. As Brazilian treatment centers are usually located in major cities, patients living in more remote areas are often unable to receive treatment at the ideal ages, resulting in more limited outcomes for such treatment.

## 2. Objective

The objective of this paper is to evaluate the profile of patients with cleft lip and palate undergoing surgery at the Cleft Palate Treatment Reference Center at the University Hospital, Rio de Janeiro Federal University, Brazil.

## 3. Methods

Data were analyzed retrospectively on 45 patients with cleft lip and/or palate, operated on consecutively by the Plastic Surgery Unit at the Clementino Fraga Filho University Hospital, Rio de Janeiro Federal University. Specific attention was paid to gender, clinical presentation, operations undergone, age of surgery, and distances between homes and the hospital.

Only patients with at least three months postoperative monitoring after primary surgery were considered in the study.

## 4. Results

Twenty-three male and 22 female patients were analyzed (Table 1).

**Figure 1:**
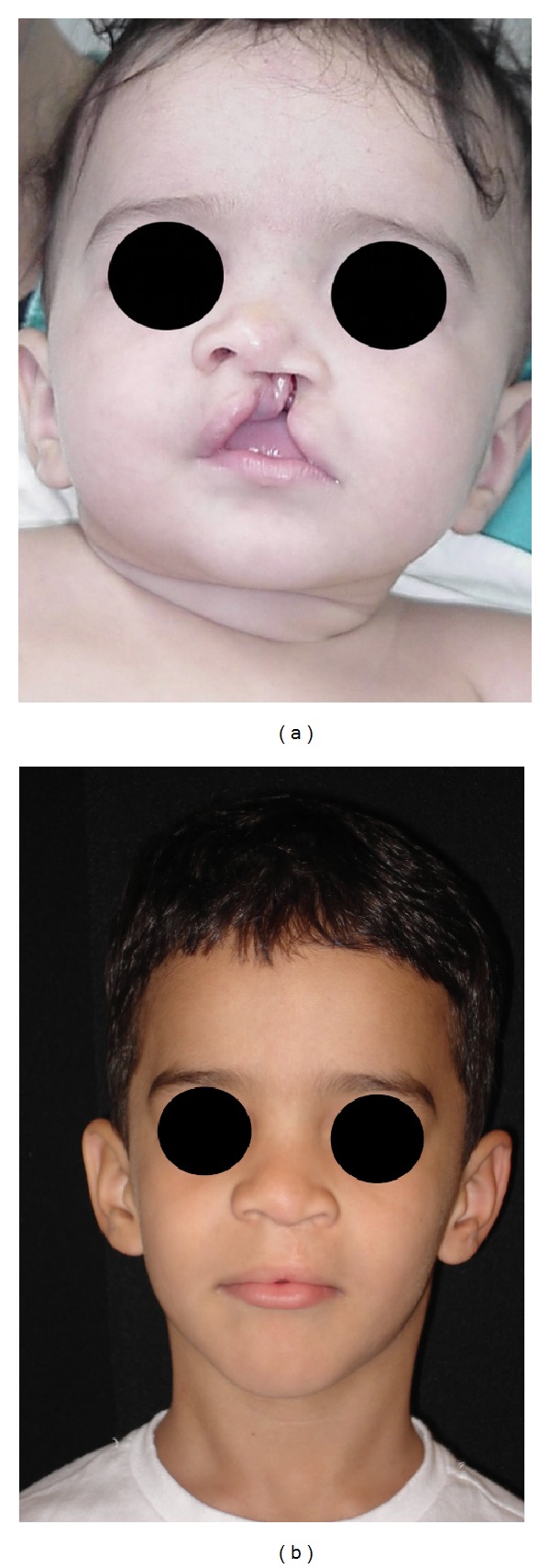
(a) Preoperative patient with complete left cleft lip and palate; (b) five years postoperative, after rhinocheiloplasty (Millard + McComb techniques).

**Figure 2:**
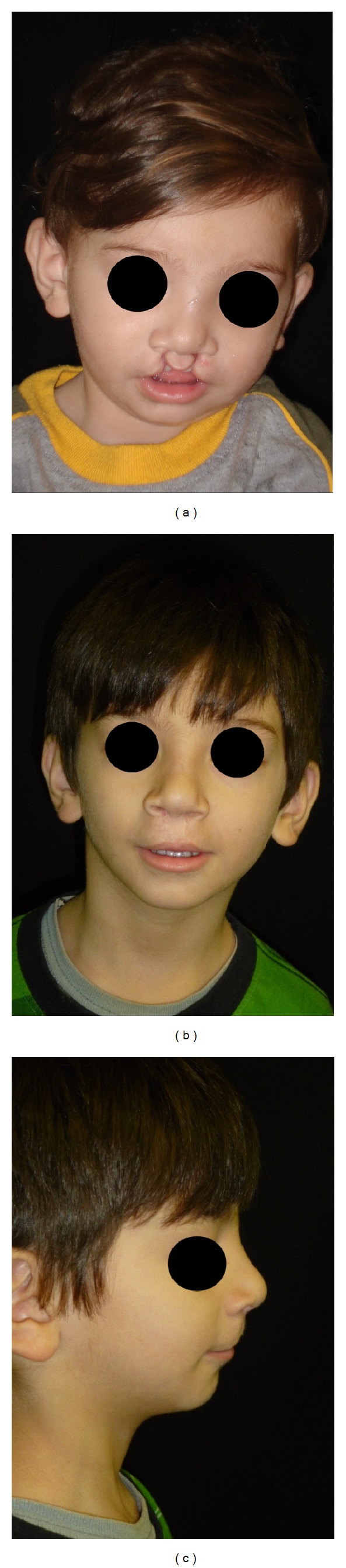
(a) Preoperative patient with complete bilateral cleft lip and palate; (b) and (c) four years postoperative, after rhinocheiloplasty, in a single operation (Mulliken technique).

Surgeries consisted of 60% palatoplasties, 38% cheiloplasties (Figures 1 and 2), and 2% cheiloplasties associated with palatoplasties (Table 2).

The average age of patients undergoing primary cheiloplasty was 9.4 months, with 74% operated on up to six months, and 26% above this age (Table 3).

With regard to the primary palatoplasties, the average age was 7.2 years, with 53% operated on between 12 and 18 months and 47% later (Table 4). The only patient undergoing combined lip and palate surgery was one year and eight months old.

Among the patients, 33% resided in the city of Rio de Janeiro, 54% lived in this state, and the remaining 13% came from elsewhere.

## 5. Discussion

Proper treatment for cleft lip and palate requires preparation, meticulous surgery, and careful postoperative monitoring besides lengthy periods of followup, involving practitioners in a wide variety of specialties, including physicians, dentists and speech therapists. The approach to these patients is complex, and in Brazil, this is normally provided at specialized centers in large cities [2]. The city of Rio de Janeiro is one of the main medical treatment hubs in Brazil, with an inflow of patients from all over the country [3].

However, facilities may be precarious or even nonexistent in poorer or more remote parts of the country. Consequently, many patients were recorded as arriving at our hospital beyond the ages rated as ideal for treatment.

In 1967, Pitanguy and Franco studied a group of 686 Brazilian patients with facial clefts. Among these, there were 84 nonoperated adults, six with cleft lip, 47 with cleft lip and palate, 14 with cleft palate, and 17 with rare facial clefts. It was noted that facial skeleton development reached a certain level of harmony, due to the balance between the growth potential of the structures and the lack of resistance normally offered by neighboring tissues. They were compared with children improperly operated at early ages, showing a final result rated as superior, due to an abundance of soft tissue that makes it easier to close combined clefts in a single operation [4].

Since then, Brazil had achieved considerable progress in terms of treating cleft lips and palates, although almost 50% of these patients are still unable to undergo surgery at the ideal time, with ratings that vary from good in major cities to extremely poor in underprivileged regions. Even today, it is not unusual to find adults who have not undergone surgery.

In our cleft palate patients, the disparity between treatment age and ideal age was significant. Only 53% of the patients were operated on at the best time, between 12 and 18 months with the average age being 7.2 years, which is far from acceptable [5–10].

For cheiloplasties, the disparity was not as great as for palatoplasties. In 74% of the cases, surgery took place up to six months of age, with an average of 9.4 months. As cleft lips are more visible, there are perhaps more pressures prompting their correction.

What contributes greatly to later presentation is the fact that many of these patients live outside the city of Rio de Janeiro (67%). This requires expenses for travel and accommodation, with days off work for caregivers, greatly hampering not only the initial presentation for surgery but also for monitoring these patients during the post-operative stage.

These geographical stumbling blocks also cause difficulties with the correct maxillary orthopedic preparation and orthodontic monitoring of these patients, which are important factors for good outcomes [11].

Many of our cleft lip and palate patients (91%) arrive with a cheiloplasty already done at another institution, before being referred to our hospital. In smaller towns, non-specialized local surgeons sometimes attempt to perform cheiloplasties, wrongly considered to be easier than palatoplasty, instead of referring these patients to a specialized Center. Consequently, most of the operations in our study consisted of palatoplasties (60%). This suboptimum conduct should be avoided, particularly as the number of cleft palate treatment centers has increased over the past decade, even in more remote areas, staffed by specialists trained to perform any procedures [2, 3]. 

Cleft lips were noted as being more frequent on the left side (62%), affecting the right side in 21% of cases, and bilateral in 17%. These findings are similar to those in the literature [5–10]. 

Recently, joint efforts by the Brazilian Government, NGOs, and groups of healthcare practitioners have managed to enlighten the public regarding treatment possibilities and organized medical programs that streamline and extend treatment facilities for this type of patient [12]. Nevertheless, there is no public health structure that encourages care-givers to seek specialized treatment at an early age for these patients, followed by the appropriate maintenance procedures. In a continent-sized country such as Brazil, where most cleft palate patients are found among the underprivileged classes, outlays on treatment may hamper and even completely prevent compliance with the protocols established by the cleft palate treatment centers.

## 6. Conclusions

The data obtained through this study show that many cleft palate patients seek treatment at ages older than those rated as ideal. The main factor behind this seems to be difficulties encountered by people living in more remote areas, who find it hard to travel to specialized centers that are generally located in major cities. Despite attempts to decentralize cleft lip and palate treatment in Brazil, adequate conditions are still not in place as required to follow the treatment protocols in a full and appropriate manner.

## Figures and Tables

**Table 1:** Distribution of clinical presentations at birth.

	Female	Male	Female + male
Unilateral left cleft lip	3	3	6 (13%)
Unilateral right cleft lip	1	2	3 (7%)
Bilateral cleft lip	1	0	1 (2%)
Unilateral left cleft lip and palate	4	8	12 (27%)
Unilateral right cleft lip and palate	2	1	3 (7%)
Complete bilateral cleft lip and palate	1	1	2 (4%)
Incomplete bilateral cleft lip and palate	0	1	1 (2%)
Complete cleft palate	4	0	4 (9%)
Incomplete cleft palate	6	7	13 (29%)
All clefts	22	23	45 (100%)

**Table 2:** Operations performed.

Palatoplasties	29 (60%)
Cheiloplasties	18 (38%)
Cheiloplasties + palatoplasties	1 (2%)

**Table 3:** Average age of patients undergoing primary cheiloplasty.

Up to six months	74%
Older than six months	26%

**Table 4:** Average age of patients undergoing primary palatoplasty.

12–18 months	53%
Older than 18 months	47%
